# Effect of task-oriented training on gross motor function, balance and activities of daily living in children with cerebral palsy: A systematic review and meta-analysis

**DOI:** 10.1097/MD.0000000000031565

**Published:** 2022-11-04

**Authors:** Weiyi Zai, Ning Xu, Wei Wu, Yueying Wang, Runfang Wang

**Affiliations:** a School of Rehabilitation Medicine, Shandong University of Traditional Chinese Medicine, Jinan, Shandong, China; b Qilu Hospital of Shandong University, Jinan, Shandong, China.

**Keywords:** activities of daily living, balance function, cerebral palsy, gross motor function, meta-analysis, task-oriented training

## Abstract

To systematically evaluate task-oriented training (TOT) on the improvement of gross motor function, balance and activities of daily living in children with cerebral palsy (CP).

**Methods:**

A number of randomized controlled trials (RCTs) of TOT in children with CP were searched from Pubmed, Cochrane Library, Web of Science, EmBase, China National Knowledge Infrastructure, Chinese Biology Medicine, Chinese Scientific Journals Database and Wanfang data from the establishment of database to March 2022. The methodological quality of the included studies was evaluated, and meta-analysis was performed by RevMan5.4 software.

**Results:**

A total of 16 studies were included in the systematic review (n = 893). Meta-analysis showed that the gross motor function measure (GMFM) (MD = 11.05, 95%CI [8.26, 13.83], *P* < .00001), dimension D (MD = 3.05, 95%CI [1.58, 4.53], *P* < .0001) of the GMFM, dimension E (MD = 7.36, 95%CI [5.88, 8.84], *P* < .00001) of the GMFM, the Berg Balance Scale (BBS) (MD = 6.23, 95%CI [3.31, 9.15], *P* < .0001), the pediatric evaluation of disability inventory (PEDI) mobile function (MD = 6.44, 95%CI [3.85, 9.02], *P* < .00001) score improved significantly in the TOT group compared with the control group.

**Conclusions:**

Current evidence shows that TOT could effectively improve gross motor function, balance and activities of daily living in children with CP. Due to the limitations of the number and quality of the included studies, the above conclusions need to be verified by more high-quality studies.

## 1. Introduction

Cerebral palsy (CP) is a group of permanent motor and postural developmental disorders that often lead to limited activity in children due to non-progressive brain damage in childbirth or after birth.^[1]^ The overall median prevalence of CP is about 2.4 per 1000 live births, which is considered to be the most common cause of severe physical disability in children.^[2,3]^ The motor development disorder caused by CP seriously affects the quality of life of children and causes a massive burden to their families and society. Improving the motor function in children with CP has always been an essential subject of rehabilitation clinical and scientific research.^[4]^ Currently, the effectiveness of neurodevelopmental therapy, traditional rehabilitation therapy (physical therapy, occupational therapy, speech therapy, etc), Botulinum toxin type A, Traditional Chinese medicine, massage, acupuncture, fumigation, and other therapeutic methods has been confirmed in clinical practice.^[5]^ Task-oriented training (TOT) is a new rehabilitation training method based on motor control theory, which emphasizes the task of simulating functional activities and pays attention to the role of the environment.^[6]^ According to individual abilities and training purposes, therapists design specific tasks or activities and guide children with CP to complete them to improve their motor skills.^[7]^ In previous studies, TOT has been proved to be an effective method for improving function in stroke patients.^[8]^ However, the application of TOT in the rehabilitation of children with CP is still in its early stages, and there is a lack of relevant evidence-based medical evidence. The aim of this review was to assess the clinical effect of TOT on improving gross motor function, balance and activities of daily living in children with CP to provide more scientific and reliable evidence for clinical practice and treatment in the future.

## 2. Data and methods

Our study was registered at the website of International Prospective Register of Systematic Reviews, and the meta-analysis was performed following the PRISMA (Preferred Reporting Items for Systematic Reviews and Meta-Analyses). The registration number is CRD42022328080. As it is a meta-analysis study, and no ethical approval was needed.

### 2.1. Inclusion criteria

#### 2.1.1. Type of studies

All the randomized controlled trials (RCTs) that TOT in children with CP were retrieved. The study language is limited to Chinese and English.

#### 2.1.2. Types of participants

Children under 18 years of age with a definite diagnosis of CP and there are no limitations on sex and race.

#### 2.1.3. Intervention measures

The control group was treated with conventional rehabilitation therapy or combined with other rehabilitation therapy (not TOT). The experimental group was given TOT alone or in combination with another rehabilitation.

#### 2.1.4. Outcome indicators

The gross motor function measure (GMFM), dimension D of the GMFM, dimension E of the GMFM, the Berg Balance Scale (BBS), and the pediatric evaluation of disability inventory (PEDI) mobile function were used to evaluate.

### 2.2. Exclusion criteria

Reviews, case reports and repeated publications; The intervention measures, outcome indicators and sample population were not eligible for inclusion; Studies with missing and incomplete relevant data; Non-RCTs; Full-text literature cannot be obtained.

### 2.3. Database and retrieval strategy

A systematic search was performed through 8 databases from the inception of the database to March 2022, including Pubmed, Cochrane Library, Web of Science, EmBase, China National Knowledge Infrastructure, Chinese Biology Medicine, Chinese Scientific Journals Database, Wanfang data, to collect the RCTs of TOT in children with CP. A combination of keywords and free words were used for retrieval, and relevant resources were also manually retrieved. The search terms included “cerebral palsy, CP, task-oriented training, repetitive task practice, task-related training, task-orientated therapy, randomized controlled trial.” Detailed search strategy is shown in Table 1.

**Table 1:** Search strategy for the PubMed database.

Number	Search term
#12	#3 AND #4 AND #11
#11	#5 OR #6 OR #7 OR #8 OR #9 OR #10
#10	Trial [Title/Abstract]
#9	Randomly [Title/Abstract]
#8	Placebo [Title/Abstract]
#7	Randomized [Title/Abstract]
#6	Controlled clinical trial [Publication Type]
#5	Randomized controlled trial [Publication Type]
#4	“Task-oriented training” [Title/Abstract] OR “repetitive task practice” [Title/Abstract] OR “task-related training” [Title/Abstract] OR “task-orientated therapy” [Title/Abstract]
#3	#1 OR #2
#2	CP (Cerebral Palsy) [Title/Abstract] OR “Cerebral Palsy,” Dystonic-Rigid[Title/Abstract] OR “Cerebral Palsies,” Dystonic-Rigid[Title/Abstract] OR “Cerebral Palsy,” Dystonic Rigid[Title/Abstract] OR “Dystonic-Rigid Cerebral Palsies” [Title/Abstract] OR “Dystonic-Rigid Cerebral Palsy” [Title/Abstract] OR “Cerebral Palsy,” Mixed[Title/Abstract] OR “Mixed Cerebral Palsies” [Title/Abstract] OR “Mixed Cerebral Palsy” [Title/Abstract] OR “Cerebral Palsy,” Monoplegic, Infantile[Title/Abstract] OR “Monoplegic Infantile Cerebral Palsy” OR “Infantile Cerebral Palsy,” Monoplegic OR “Cerebral Palsy,” Quadriplegic, Infantile OR “Quadriplegic Infantile Cerebral Palsy” [Title/Abstract] OR “Infantile Cerebral Palsy,” Quadriplegic[Title/Abstract] OR “Cerebral Palsy,” Rolandic Type[Title/Abstract] OR “Rolandic Type Cerebral Palsy” [Title/Abstract] OR “Cerebral Palsy,” Congenital[Title/Abstract] OR “Congenital Cerebral Palsy” [Title/Abstract] OR “Little Disease” [Title/Abstract] OR “Little’s Disease” [Title/Abstract] OR “Spastic Diplegia” [Title/Abstract] OR “Diplegias,” Spastic[Title/Abstract] OR “Spastic Diplegias” [Title/Abstract] OR “Diplegia,” Spastic[Title/Abstract] OR “Monoplegic Cerebral Palsy” [Title/Abstract] OR “Cerebral Palsies,” Monoplegic[Title/Abstract] OR “Cerebral Palsy,” Monoplegic[Title/Abstract] OR “Monoplegic Cerebral Palsies” [Title/Abstract] OR “Cerebral Palsy,” Athetoid[Title/Abstract] OR “Athetoid Cerebral Palsy” [Title/Abstract] OR “Cerebral Palsies,” Athetoid[Title/Abstract] OR “Cerebral Palsy,” Dyskinetic[Title/Abstract] OR “Cerebral Palsies,” Dyskinetic[Title/Abstract] OR “Dyskinetic Cerebral Palsy” [Title/Abstract] OR “Cerebral Palsy,” Atonic[Title/Abstract] OR “Atonic Cerebral Palsy” [Title/Abstract] OR “Cerebral Palsy,” Hypotonic[Title/Abstract] OR “Hypotonic Cerebral Palsies” [Title/Abstract] OR “Hypotonic Cerebral Palsy” [Title/Abstract] OR “Cerebral Palsy, Diplegic,” Infantile[Title/Abstract] OR “Diplegic Infantile Cerebral Palsy” [Title/Abstract] OR “Infantile Cerebral Palsy,” Diplegic[Title/Abstract] OR “Cerebral Palsy,” Spastic[Title/Abstract] OR “Spastic Cerebral Palsies” [Title/Abstract] OR “Spastic Cerebral Palsy” [Title/Abstract]
#1	Cerebral palsy [MeSH]

### 2.4. Data collection and extraction

EndnoteX9 software was used for study information management, and duplicate studies were excluded. Two investigators read the titles and abstracts of the papers and excluded obvious nonconformities. Then the investigators carefully examined the full text and included trials that met the inclusion criteria according to the inclusion and exclusion criteria established earlier. The study screening process was carried out by 2 researchers independently, and the results were cross-checked. If there was any disagreement, a third arbitrator was involved.

The extracted data included: basic information of the included study (first author, publication time), basic characteristics of the study subjects (sample size, type of motor impairment, gender, age), intervention methods, intervention frequency, intervention cycle, main data of outcome indicators and key elements of bias risk assessment.

### 2.5. Quality of evidence

The risk of bias for RCTs was assessed by the Cochrane Handbook 5.1.0. Each study was objectively evaluated as “low risk”, “high risk”, or “unclear risk” based on the 7 domains of quality criteria as follows: random sequence generation; allocation concealment; blinding of participants and personnel; blinding of outcome assessors; incomplete outcome data; selective reporting; other bias. The risk of bias was evaluated by 2 researchers independently, and the results were cross-checked. In addition, disagreements were resolved by a third arbitrator.

### 2.6. Statistical analysis

RevMan5.4 software was used for meta-analysis of outcome indicators. The data types of outcome indexes of this meta-analysis were all continuous data, and the mean difference (MD) and 95% confidence interval (CI) with fixed or random effect models will be used for calculation. *χ*^2^ test (α = 0.05) was used to analyze the heterogeneity among the results and combined with *I*² to quantitatively judge the heterogeneity. A random effects model was chosen if high heterogeneity was observed (*P* < .05, *I*^2^ > 50%). Otherwise, a fixed effects model was adopted. If there is significant clinical heterogeneity, subgroup analysis or sensitivity analysis are used to treat it, or descriptive analysis is performed only. The reported bias will be shown by the funnel plot.

## 3. Results

### 3.1. Study identification

**Figure 1.**
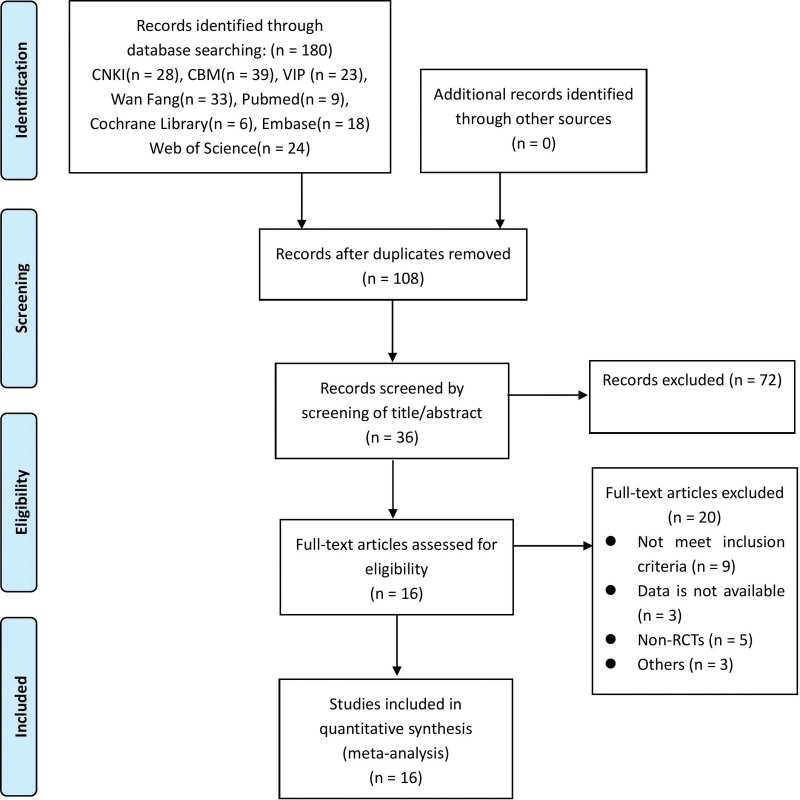
Flow chart of the search and study selection process.

A total of 180 studies were obtained, including 123 Chinese studies and 57 English studies. 108 studies remained after eliminating duplicate studies. 36 studies remained after reading the titles and abstracts and excluding those not meeting inclusion criteria. 16 studies were finally included after reviewing the full text. A flowchart of the retrieval process is shown in Figure 1.

### 3.2. Characteristics of included studies

16 studies were included in this study, including 12 Chinese studies^[9–20]^ and 4 English studies.^[21–24]^ The sample size of children with CP ranged from 10 to 123 cases, including 448 cases in the experimental group and 445 cases in the control group. The baseline data of included subjects were comparable. The control group received conventional rehabilitation therapy (traditional physical rehabilitation training, facilitation techniques, traditional physical and occupational therapy, etc) or combined with other rehabilitation therapy (not TOT). On this basis, the experimental groups were given TOT with different frequencies. The treatment duration ranged from 4 weeks to 4 months. The basic data included in the study are shown in Table 2.

**Table 2:** Basic characteristics of the included studies.

Study	Age (yr)	Sample		Intervention		Training frequency (TOT)	Treatment duration	Outcome indicator
		T	C	T	C			
Ko EJ^[21]^ 2020	4–7.5	9	9	TOT	Traditional PT and OT	60 min/time, 2 times/wk	8 wk	③④⑥
HK Han^[22]^ 2016	7–15	12	12	Conventional rehabilitation + TOT	Conventional rehabilitation	30 min/d, 5 d/wk	4 wk	②
Salem Y^[23]^ 2009	4–12	5	5	TOT	Conventional PT	2 times/wk	5 wk	③④
Wang GX^[9]^ 2017	3–12	25	24	Conventional rehabilitation + TOT	Conventional rehabilitation	50 min/d, 5 d/wk	12 wk	③④
Zhang HX^[10]^ 2021	3–8	48	47	Conventional rehabilitation + TOT	Conventional rehabilitation	40 min/d, 5 d/wk	3 mo	③④
Zhang CY^[11]^ 2014	2–12	25	25	Conventional rehabilitation + TOT	Conventional rehabilitation	10~15 min/d, 5 d/wk	3 m	⑤
Pang W^[12]^ 2016	3–12	20	20	Conventional rehabilitation + TOT	Conventional rehabilitation	40 min/d, 5 d/wk	3 m	③④
Li Y^[13]^ 2019	2–9	41	41	Traditional PT + TOT	Traditional PT	20 min/d	3 m	②⑥
Li X^[14]^ 2015	3–9	30	30	Conventional rehabilitation + TOT	Conventional rehabilitation	40 min/d, 5 d/wk	3 m	③④⑥
Fan TL^[15]^2019	6–12	22	23	Conventional rehabilitation + TOT	Conventional rehabilitation	40 min/d, 5 d/wk	120 d	④⑤⑥
Cheng K^[16]^ 2010	1–3	25	25	Facilitation techniques + TOT	Facilitation techniques	40 min/d, 5 d/wk	3 m	①
Sah AK^[24]^ 2019	7–15	22	22	TOT	Conventional PT	60 min/d, 6 d/wk	6 wk	②
Zhang WD^[17]^ 2021	3–6	62	61	Conventional rehabilitation + Biofeedback training + TOT	Conventional rehabilitation + Biofeedback training	40 min/d, 6 d/wk	4 m	③④⑤
Lyu YB^[18]^ 2020	2–6	40	40	Conventional rehabilitation + TOT	Conventional rehabilitation	/	/	②⑤
Chen C^[19]^ 2009	1–3	20	20	Facilitation techniques + TOT	Facilitation techniques	40 min/d	3 m	①
Zhao XY^[20]^ 2020	3–7	42	41	Conventional rehabilitation + TOT	Conventional rehabilitation	30 min/d,5 d/wk	8 wk	⑤

①GMFM-66, ②GMFM-88, ③dimension D of the GMFM, ④dimension E of the GMFM, ⑤BBS, ⑥PEDI mobile function.

BBS = the Berg Balance Scale, C = control group, GMFM-66 = the gross motor function measure-66, GMFM-88 = the gross motor function measure-88, OT = occupational therapy, PEDI = the pediatric evaluation of disability inventory, PT = physical therapy, T = treatment group, TOT = task-oriented training.

### 3.3. Quality of evidence

**Figure 2.**
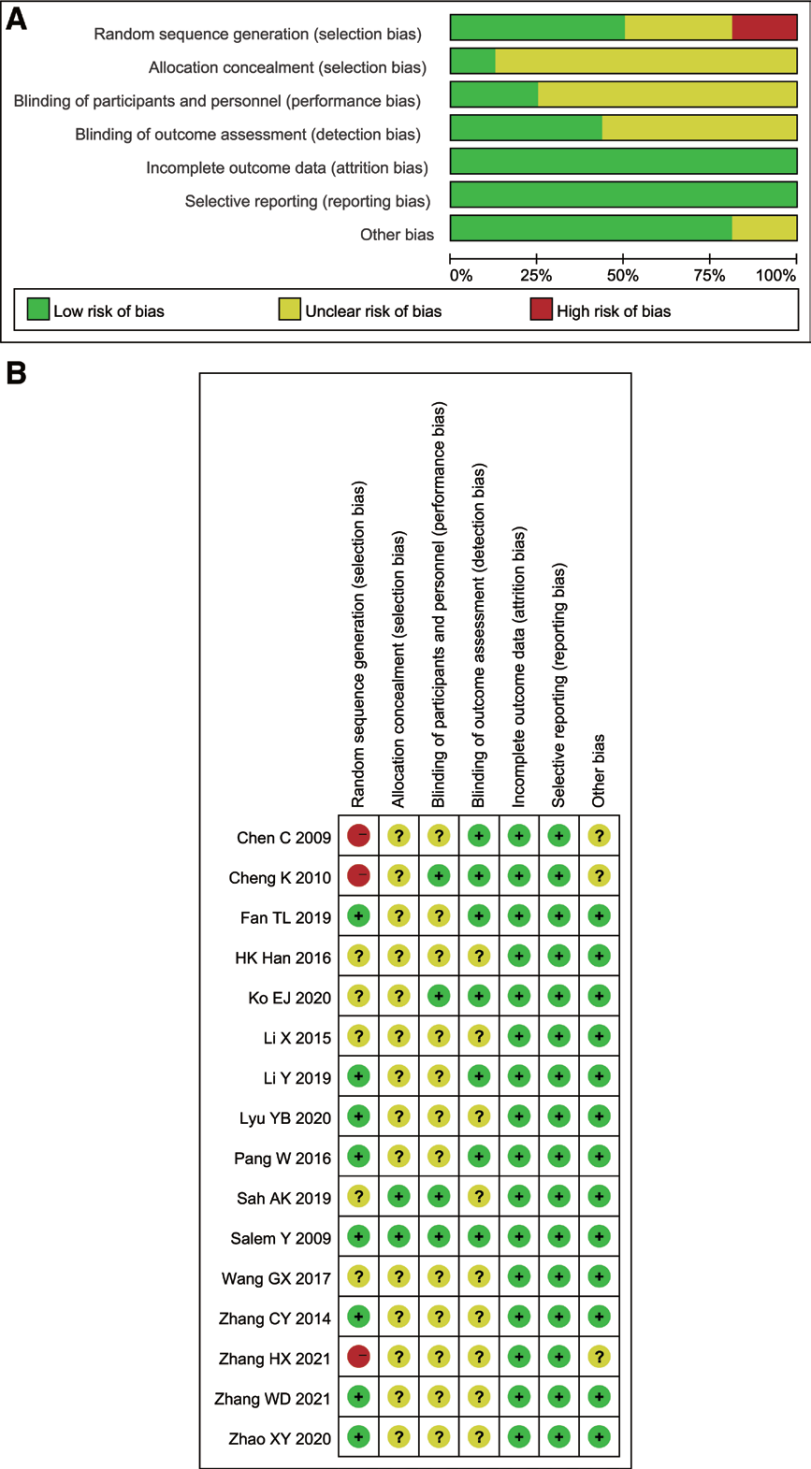
Risk of bias graph and summary. (A) Risk of bias graph. (B) Risk of bias summary.

Cochrane Bias risk Assessment tool was used to evaluate the quality of the included studies, of which 1 study^[23]^ was rated as A and 15 studies^[9–22,24]^ were rated as B. Total 8 studies^[11–13,15,17,18,20,23]^ clearly described the generation process of random sequences, 5 studies^[9,14,21,22,24]^ only mentioned random, and 3 studies^[10,16,19]^ adopted nonrandom methods. Total 2 studies^[23,24]^ reported the hidden process of allocation, and the rest were not mentioned. Blinding was applied in 8 studies,^[12,13,15,16,19,21,23,24]^ but most were single-blind. All the research data were complete and all pre-designed indicators were reported. No other biases were found in 13 studies.^[9,11–15,17,18,20–24]^ Risk of bias assessment details is provided in Figure 2.

### 3.4. Meta-analysis results

#### 3.4.1. GMFM

6 studies^[13,16,18,19,22,24]^ used GMFM score as an outcome indicator, of which 2 studies^[16,19]^ used GMFM-66 score and 4 studies^[13,18,22,24]^ used GMFM-88 score, including 320 children with CP. There was a low heterogeneity (*χ*^2^ = 5.49, *P* = .36, *I*^2^ = 9%), so a fixed effects model was adopted. The results of meta-analysis showed that GMFM score in the TOT group was higher than that in the control group, and the difference was statistically significant (MD = 11.05, 95%CI [8.26, 13.83], *P* < .00001).

**Figure 3.**
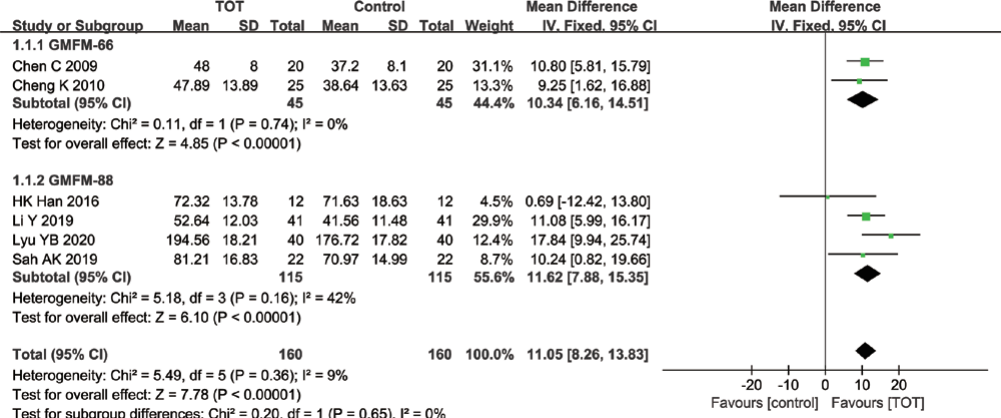
Forest plot of the effect of TOT on GMFM. GMFM = the gross motor function measure, TOT = task-oriented training.

Subgroup analysis of GMFM score was performed according to the different version of the scale. The results showed that GMFM-66 score in the TOT group was significantly higher than that in the control group (MD = 10.34, 95%CI [6.16, 14.51], *P* < .00001), and GMFM-88 score in the TOT group was significantly higher than that in the control group (MD = 11.62, 95%CI [7.88, 15.35], *P* < .00001). All the above results prove that TOT can improve gross motor function in children with CP (Fig. 3).

#### 3.4.2. Dimension d of the GMFM

**Figure 4.**
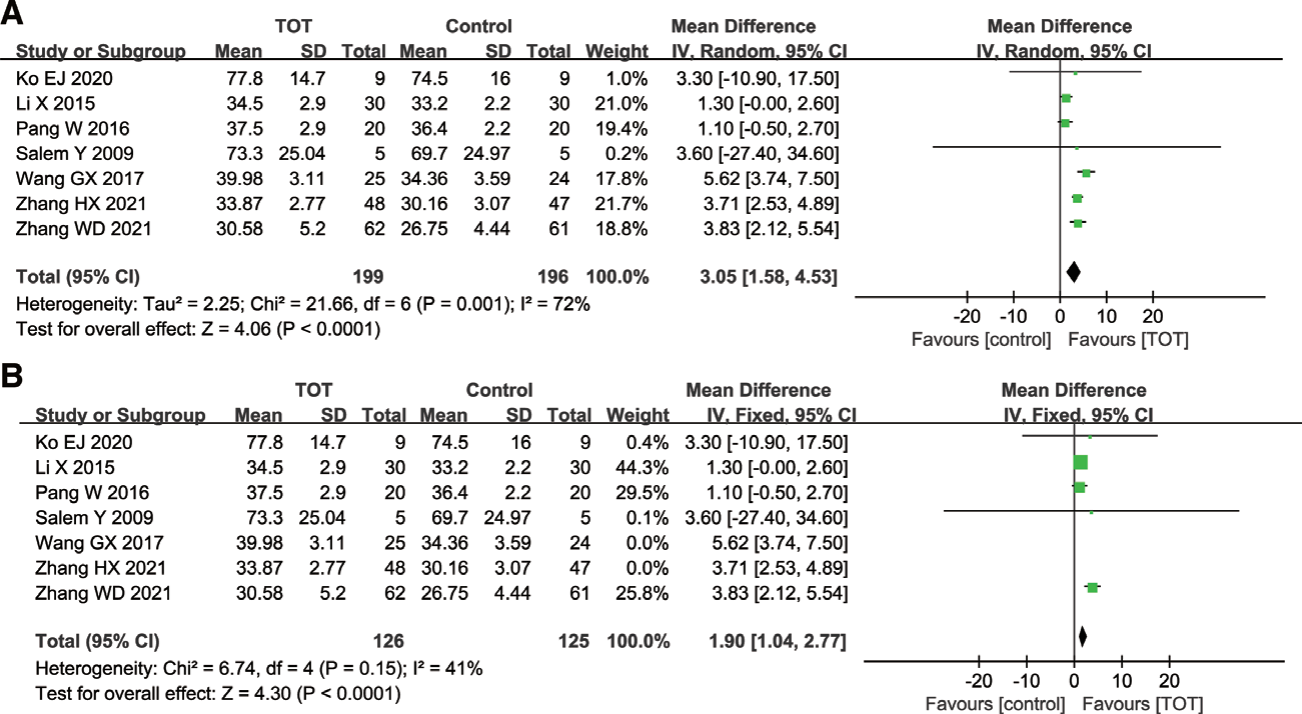
Forest plot of the effect of TOT on dimension D of the GMFM and dimension D of the GMFM after sensitivity analysis. (A) Forest plot of the effect of TOT on dimension D of the GMFM. (B) Forest plot of the effect of TOT on dimension D of the GMFM after sensitivity analysis. GMFM = the gross motor function measure, TOT = task-oriented training.

Total 7 studies^[9,10,12,14,17,21,23]^ used dimension D of the GMFM score as an outcome index, including 395 children with CP. There was a high heterogeneity among the studies (*χ*^2^ = 21.66, *P* = .001, *I*^2^ = 72%), so a random effects model was used. The results of meta-analysis showed that dimension D of the GMFM score in the TOT group was significantly higher than that in the control group (MD = 3.05, 95%CI [1.58, 4.53], *P* < .0001), which proved that TOT was helpful to improve the standing function in children with CP. Due to a high heterogeneity, the sensitivity analysis was performed. After excluding the studies of Wang GX^[9]^ and Zhang HX,^[10]^ the heterogeneity among the studies decreased significantly (*χ*^2^ = 6.74, *P* = .15, *I*^2^ = 41%) (Fig. 4).

#### 3.4.3. Dimension e of the GMFM

**Figure 5.**
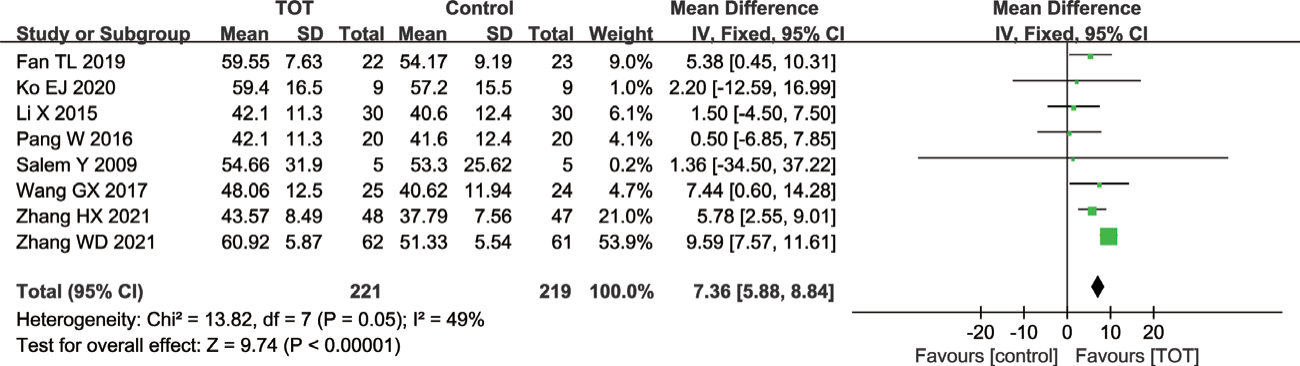
Forest plot of the effect of TOT on dimension E of the GMFM. GMFM = the gross motor function measure, TOT = task-oriented training.

Total 8 studies^[9,10,12,14,15,17,21,23]^ used dimension E of the GMFM score as an outcome measure, including 440 children with CP. A fixed effects model was used because of a low heterogeneity (*χ*^2^ = 13.82, *P* = .05, *I*^2^ = 49%). The results of meta-analysis showed that dimension E of the GMFM score in the TOT group was higher than that in the control group (MD = 7.36, 95%CI [5.88, 8.84], *P* < .00001), which proved that TOT could improve the walking function in children with CP (Fig. 5).

#### 3.4.4. BBS

**Figure 6.**
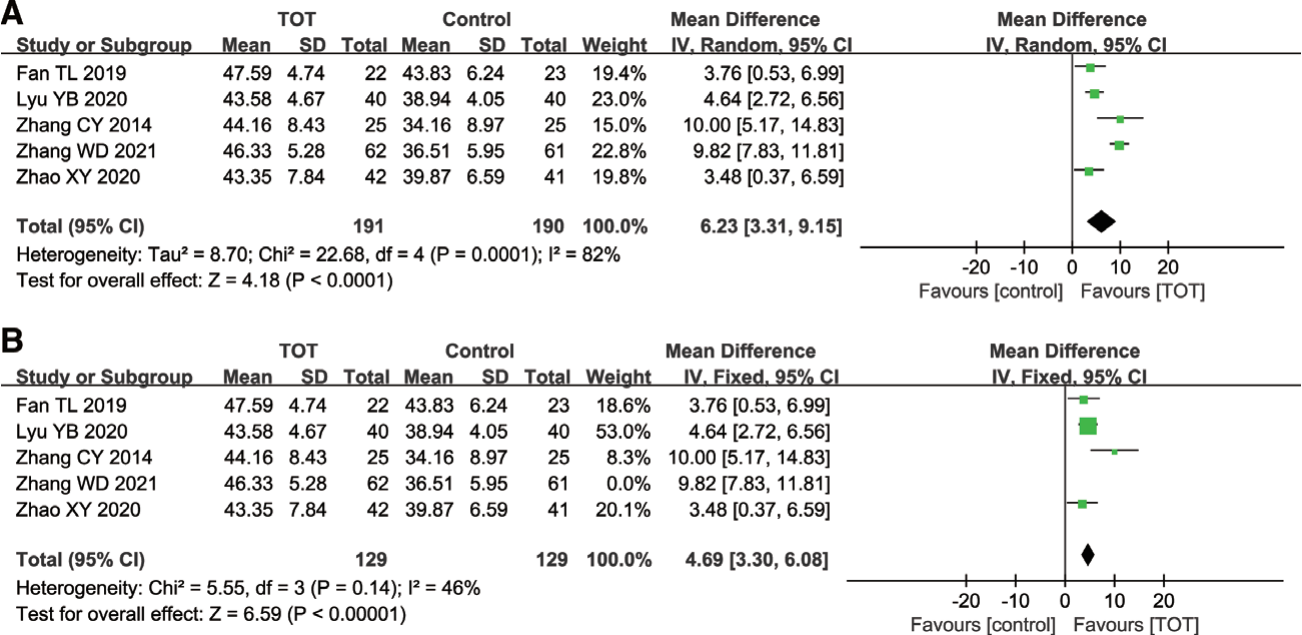
Forest plot of the effect of TOT on BBS and BBS after sensitivity analysis. (A) Forest plot of the effect of TOT on BBS. (B) Forest plot of the effect of TOT on BBS after sensitivity analysis. BBS = the Berg Balance Scale, TOT = task-oriented training.

Total 5 studies^[11,15,17,18,20]^ used the BBS score as an outcome index, including 381 children with CP. The heterogeneity among the studies was high (*χ*^2^ = 22.68, *P* = .0001, *I*^2^ = 82%), so a random effects model was chosen. The results of meta-analysis showed that the score of BBS in the TOT group was higher than that in the control group (MD = 6.23, 95%CI [3.31, 9.15], *P* < .0001), which proved that TOT helped improve the balance function in children with CP. Due to a high heterogeneity, the sensitivity analysis was performed. After excluding the study of Zhang WD,^[17]^ the heterogeneity among the studies decreased significantly (*χ*^2^ = 5.55, *P* = .14, *I*^2^ = 46%) (Fig. 6).

#### 3.4.5. PEDI mobile function

**Figure 7.**
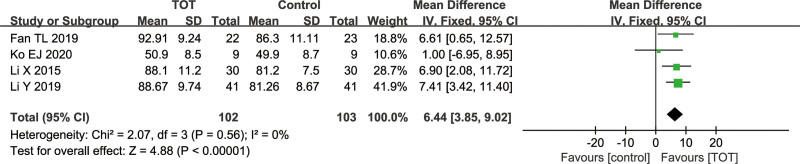
Forest plot of the effect of TOT on PEDI mobile function. PEDI = the pediatric evaluation of disability inventory, TOT = task-oriented training.

Total 4 studies^[13–15,21]^ used PEDI mobility function score as an outcome index, including 205 children with CP. There was a low heterogeneity among the studies (*χ*^2^ = 2.07, *P* = .56, *I*^2^ = 0%), so a fixed effects model was used. The results of meta-analysis showed that PEDI mobility function score in the TOT group was higher than that in the control group (MD = 6.44, 95%CI [3.85, 9.02], *P* < .00001), indicating that TOT can improve the ability of daily living activities in children with CP (Fig. 7).

#### 3.4.6. Publication bias

**Figure 8.**
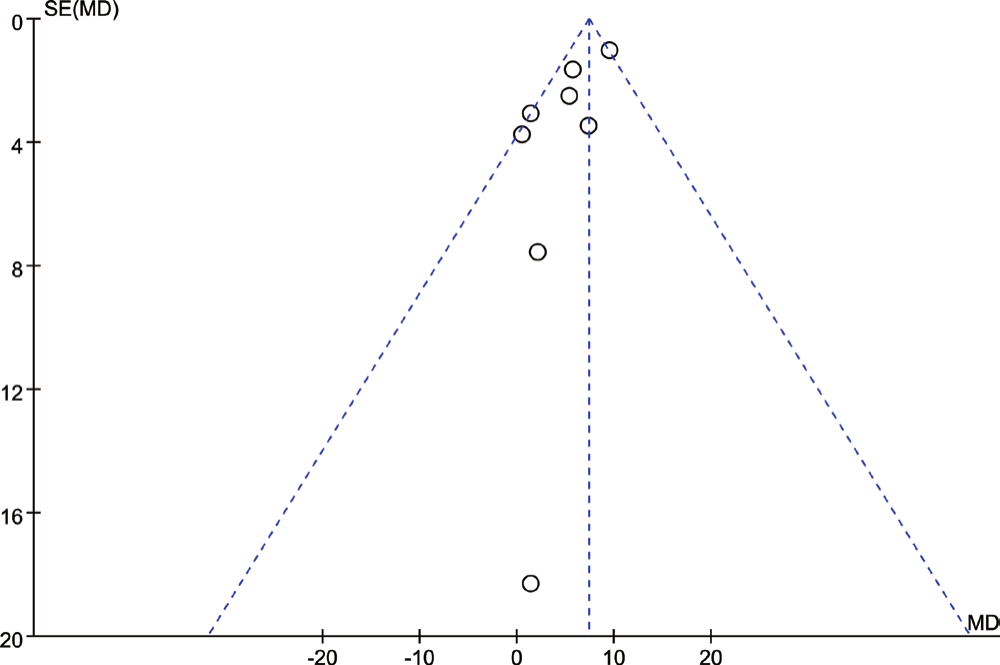
Funnel plot of dimension E of the GMFM. GMFM = the gross motor function measure.

The “dimension E of the GMFM” was selected as an indicator to analyze the publication bias of the included studies, and the results showed that studies were mainly concentrated in the upper 1/3 of the funnel plot, indicating that there was little possibility of significant publication bias (Fig. 8).

## 4. Discussion

CP is a neurodevelopmental disease characterized by abnormal muscle tone, movement and motor skills, which seriously limits children’s activity and social participation.^[25]^ Although modern medical technology has made significant progress, the rehabilitation of children with cerebral palsy remains a huge challenge. Currently, the existing rehabilitation methods in clinical practice mainly aim at motor problems, such as abnormal muscle tone, abnormal reflexes, abnormal movement patterns, etc., while functional movement is often ignored.^[26]^ The International Classification of Functioning, Disability and Health (ICF) recommends that rehabilitation should focus on activity and participation limitations.^[27]^ TOT attaches importance to the interaction between individuals, tasks and the environment in which tasks are performed and emphasizes the establishment of “functional tasks.” Children with CP can actively try to solve problems in functional tasks, adapt to environmental changes, and apply the functions acquired in training to the real environment.^[28]^

Neural plasticity is a piece of strong evidence that TOT can improve the function in children with CP. The brain can reconstruct cortical motor maps by constantly establishing new neural connections and neural networks.^[29,30]^ It is important to note that adaptive cortical recombination in both intact and injured central nervous system (CNS) is not induced by generic use or activation but requires task specific training protocols.^[31]^ The combination of a rich environment and task-specific rehabilitation can enhance the plasticity of inherent neurons in non-injured and functionally connected brain regions and achieve the result of improving function.^[32]^ The actual operation of TOT involves the brain’s judgment of information and the innervation of nerves to motion. After repeated practice and constant modes adjustment, an optimized neural network and motion program can be formed to innervate relevant muscles to complete specific tasks. If the upper limb is flexing and extending without a particular goal, the integration and input of the above comprehensive information will be lost, and the motion mechanics characteristics will become an empty joint activity.^[33]^ TOT is a controlled exercise training emphasizing the active participation of children with CP, which has prominent advantages in the rehabilitation of children with CP.

GMFM is an international index to evaluate the gross motor development in children with CP, with 2 versions: GMFM-66 and GMFM-88. The higher GMFM scores indicate an excellent effect on children with CP. The subgroup analysis of GMFM outcome indicators showed no significant difference in the score of GMFM-66 and GMFM-88 between the 2 groups, suggesting that TOT can improve gross motor function in children with CP. Dimension D of the GMFM is mainly used to evaluate the standing level of children. Meta-analysis results showed that TOT could improve the standing function in children with CP, but there was a high heterogeneity. The sensitivity analysis showed that the heterogeneity came from Wang GX^[9]^ and Zhang HX.^[10]^ However, the specific reasons for a high heterogeneity have not been found, which may be due to methodological deficiencies in both studies. These 2 studies have unclear risk and high risk in the random sequence generation, allocation concealment and blinding. Dimension E of the GMFM is used to evaluate the walking function of the children. Meta-analysis showed that TOT could improve the walking function in children with CP.

BBS is mainly used to evaluate the recovery of balance function in children with CP. Meta-analysis results showed that TOT could improve the balance function in children with CP, but the heterogeneity was high. Through sensitivity analysis, it is concluded that heterogeneity comes from the study of Zhang WD,^[17]^ which may be related to different intervention measures. In this study, the intervention measures in the experimental group were a combination of 2 treatments and TOT.

PEDI mobility function is used to evaluate the mobility of daily life in children with CP. Meta-analysis results showed that TOT could effectively improve limb motor function and the ability to independently perform daily activities in children with CP, and there was a low heterogeneity among the studies. GMFM can only reflect the best completion of motor function in children with CP after receiving corresponding instructions in a specific assessed environment. In contrast, PEDI focuses on the evaluation of the level of activity and participation in the ICF framework, which can reflect the performance of motor function in children with CP in daily life.^[34,35]^ The above results proved that TOT could improve the motor function and activities of daily living in children with CP, enabling them to better integrate into school and society.

In the retrieval process, researchers found that many studies combined with other treatment methods basis on TOT, such as head low frequency electrical stimulation,^[36]^ biofeedback training,^[28]^ and hydrotherapy,^[37]^ had better efficacy than TOT alone in improving the motor function in children with CP. In addition, emphasizing the participation of families of children with CP in the treatment of TOT can further enhance their confidence in rehabilitation and contribute to completing the task.^[38,39]^ These results suggest that further research should focus on the combination of TOT with other therapies and further research on “home-based” task-oriented exercise to play a more significant role in the rehabilitation of children with CP.

The study found that the 16 included studies had the following deficiencies: The subjects of the RCTs were not completely uniform in the types of disease, age, treatment plan, etc., leading to a high heterogeneity among the studies. The methodological representation of the included studies was vague, some of the studies did not specify the randomization method, and most did not mention the allocation concealment and blinding, leading to a particular publication bias. There are many domestic and foreign studies on improving gross motor function, balance function and activities of daily living in children with CP, but the evaluation indicators are not the same. In this study, only GMFM, BBS, and PEDI mobile function were selected, and the limited number of included studies may affect the evaluation results to some extent.

In conclusion, current evidence suggests that TOT can significantly improve gross motor function, balance function, and activities of daily living in children with CP compared with conventional rehabilitation techniques. However, due to the limited number and quality of included studies, more high-quality RCTs are needed to provide a more scientific basis for applying TOT in clinical practice.

## Author contributions

**Conceptualization:** Weiyi Zai, Ning Xu, Wei Wu.

**Data curation:** Weiyi Zai, Ning Xu, Wei Wu.

**Funding acquisition:** Ning Xu, Wei Wu.

**Methodology:** Weiyi Zai, Ning Xu, Wei Wu, Runfang Wang.

**Resources:** Weiyi Zai, Yueying Wang, Runfang Wang.

**Software:** Weiyi Zai, Yueying Wang, Runfang Wang.

**Visualization:** Ning Xu.

**Writing – original draft:** Weiyi Zai.

**Writing – review & editing:** Ning Xu, Wei Wu.
